# *Toxins*: State of the Journal Report, 2017

**DOI:** 10.3390/toxins9020055

**Published:** 2017-02-04

**Authors:** Chao Xiao, Vernon L. Tesh

**Affiliations:** 1 *Toxins* Editorial Office, MDPI AG, St. Alban-Anlage 66, 4052 Basel, Switzerland; toxins@mdpi.com; 2 Department of Microbial and Molecular Pathogenesis, Medical Research and Education Building, Room 3002, College of Medicine, Texas A&M University System Health Science Center, 8447 State Highway 47, Bryan, TX 77807, USA; TESH@medicine.tamhsc.edu

On behalf of the *Toxins* editorial team, we are happy to report that the impact factor for *Toxins* for 2015 is 3.571, 5-year impact factor: 3.942, which places the journal at a ranking of 16th out of 89 journals covering the field of toxicology. Both the number of submissions and the number of articles published (Figure 1) continued an upward trend in 2016. The number of citations and article (pdf) downloads also increased (Figure 2 and Figure 3). Our acceptance rate for 2016 was 51.6% and the median time from first submission to publication was 57 days, a first decision was provided to authors approximately 20 days after submission. Collectively, the journal statistics suggest that *Toxins* continues to be an important resource for investigators in the field of toxinology. We are grateful to our colleagues for your support of the journal by the submission of outstanding papers for review, and for taking time from your own work to carefully and thoughtfully review manuscripts submitted to *Toxins*.

In 2016, we implemented double blind peer-review for all papers submitted to the journal. In this procedure, authors’ names are not known to the reviewers and reviewers’ names are not known to the authors until publication of the paper. We feel this procedure reduces bias in the review process without significantly adding time from receipt of a manuscript to posting the accepted paper on the *Toxins* webpage. We have paid special attention to the nomenclature of toxins, including the evaluation of whether venom peptide toxins are correctly described and identified (see Instructions to Authors at http://www.mdpi.com/journal/toxins/instructions). In 2017, a historical perspective and guidelines for Botulinum Neurotoxin subtype nomenclature by Michael W. Peck et al. (http://www.mdpi.com/2072-6651/9/1/38), as well as an interview with Jay Fox and José Maria Gutiérrez on snake venom metalloproteinases (http://www.mdpi.com/2072-6651/9/1/33) have been published. We would welcome additional submissions on clarification of toxin nomenclature. 

In 2016, we published five comments on previously published papers and welcome further comments, recommending *Toxins* as a forum for civil discourse on issues impacting toxinology. The two most accessed papers from 2016 are two original research articles: The Snake with the Scorpion’s Sting: Novel Three-Finger Toxin Sodium Channel Activators from the Venom of the Long-Glanded Blue Coral Snake (*Calliophis bivirgatus*) by Bryan G. Fry, et al., and Cyanobacterial Neurotoxin BMAA and Mercury in Sharks by Deborah C. Mash, et al.

In 2016, we also published two books: Enterotoxins: Microbial Proteins and Host Cell Dysregulation, edited by Dr. Teresa Krakauer, and Harmful Algal Blooms (HABs) and Public Health: Progress and Current Challenges, edited by Drs. Lesley V. D’Anglada, Elizabeth D. Hilborn, and Lorraine C. Backer. The e-book versions may be downloaded free of charge from the *Toxins* webpage, and printed versions can be ordered at http://www.mdpi.com/books/library or Applebook and Amazon. We are always open to discuss your proposals for new book topics in 2017. We sponsored six conferences in 2016 and we published reports on presentations from two conferences: the 5^th^ International Symposium on Mycotoxins and Toxigenic Moulds: Challenges and Perspectives (MYTOX) and the Hinxton Retreat on Mechanisms to Reverse the Public Health Neglect of Snakebite Victims. We look forward to continuing to support scientific presentations in 2017 and please keep the journal in mind as a resource for communicating meeting presentations to a broader audience. In 2017, we provided travel award support for three post-doctoral fellows/senior graduate students to present their work at international toxinology conferences, and further details of the winners are announced at http://www.mdpi.com/journal/toxins/awards. We congratulate Dr. Marco Pirazzini, Dr. Natalie Saez and Ms. Rachel A. Miller on winning the 2017 *Toxins* Travel Awards.

We gratefully acknowledge the outstanding service of two out-going Section Editors, Dr. Richard A. Manderville, Section Editor-in-Chief for Mycotoxins, and Dr. John P. Berry, Section Editor-in-Chief for Marine and Freshwater Toxins. The numbers of excellent papers on mycotoxins and marine and freshwater toxins published during the tenure of Dr. Manderville and Dr. Berry attest to their hard work as Section Editors in maintaining high standards for the readers of *Toxins*. We are also grateful to Dr. Sarah De Saeger and Dr. Vitor Vasconcelos who will replace Dr. Manderville and Dr. Berry as Section Editors-in-Chief for Mycotoxins, and Marine and Freshwater Toxins, respectively (Figure 4). Dr. De Saeger is Director of the Laboratory for Food Analysis and Professor in the Department of Bioanalysis, Faculty of Pharmaceutical Sciences, Ghent University, Belgium. She has published nearly 300 papers on the impact of mycotoxins on human and animal health, the measurement of biomarkers of exposure to mycotoxins, and mycotoxin occurrence in foods and feeds. Dr. Vitor Vasconcelos is Professor, Faculty of Sciences, University of Porto, Portugal, Director in the Interdisciplinary Center of Marine and Environmental Research (CIIMAR) and Head of the Group on Blue Biotechnology and Ecotoxicology (LEGE lab). He has published nearly 300 papers in the areas of toxicology and biotechnology. Dr. Vasconcelos’ main research efforts focus on the diversity, intoxication dynamics and environmental and human health risks of cyanobacterial toxins. Dr. Vasconcelos also studies emergent marine toxins, including tetrodotoxins, ciguatoxins, palitoxins and their analogues. The readers of *Toxins* are indeed fortunate that such prestigious scientists volunteer their time and effort to serve investigators in the toxinology field.

Finally, take a look at the new *Toxins* webpage design. The site has been redesigned to provide more information on the homepage, including fast access to the latest articles, access to recent articles of high interest, information on upcoming conferences, and quick links to Special Issues, topical collections, conference reports and books. Go to “Journal Browser” and click on Volume 8, where you will see we have begun designing volume covers for each monthly issue. You may find your favorite venomous critter or toxigenic microorganism on the cover! In 2017, we will also start posting research job adverts on the journal homepage. You are welcome to contact us for posting your vacancies.

In closing, it has been a great year at *Toxins*. Your support of the journal has been fantastic. It is through your efforts that the journal continues to be a useful resource for investigators, students and the lay public. We are greatly appreciative of your hard work. By all means, do not hesitate to contact us with ideas, criticisms, words of wisdom, etc., on how to better serve the toxinology research community.

## Figures and Tables

**Figure 1:**
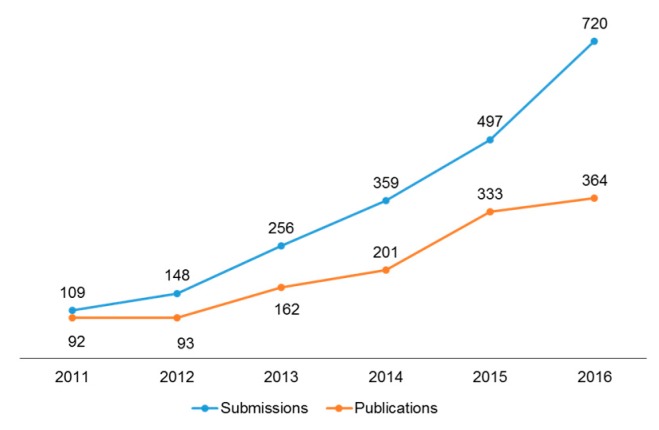
Growth in number of submissions and publications since 2011.

**Figure 2:**
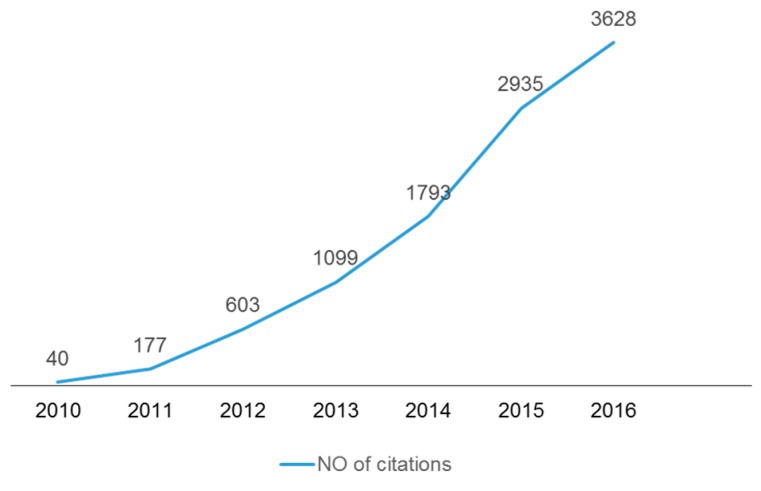
Growth in citations of *Toxins* papers since 2010.

**Figure 3:**
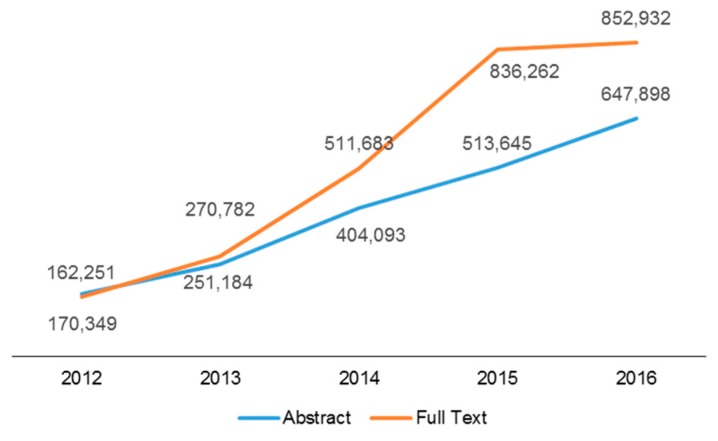
Growth in abstract and full text page views through MDPI.

**Figure 4:**
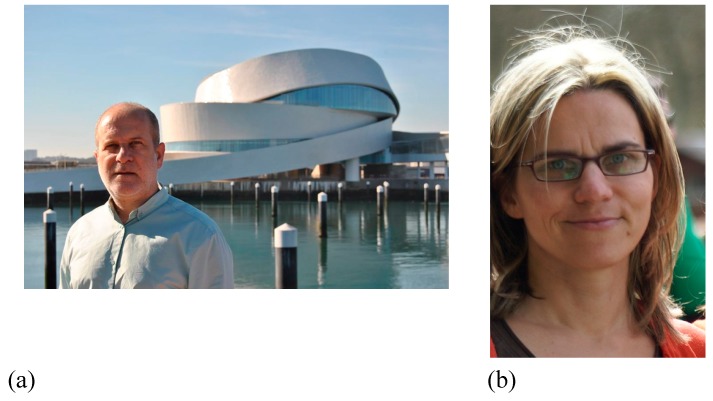
(**a**) Dr. Vitor Vasconcelos; (**b**) Dr. Sarah De Saeger.

